# C-Terminal Tensin-Like Protein Is a Novel Prognostic Marker for Primary Melanoma Patients 

**DOI:** 10.1371/journal.pone.0080492

**Published:** 2013-11-07

**Authors:** Cecilia Sjoestroem, Shahram Khosravi, Guohong Zhang, Magdalena Martinka, Gang Li

**Affiliations:** 1 Department of Dermatology and Skin Science, Vancouver Coastal Health Research Institute, University of British Columbia, Vancouver, British Columbia, Canada; 2 Department of Pathology, Vancouver General Hospital, Vancouver, British Columbia, Canada; University of Connecticut Health Center, United States of America

## Abstract

**Background:**

C-terminal tensin-like protein (Cten) is a focal adhesion protein originally identified as a tumor suppressor in prostate cancer. It has since been found to be overexpressed and function as an oncogene in numerous other cancers, but the expression status of Cten in melanoma is still unknown.

**Methods:**

Using tissue microarrays containing 562 melanocytic lesions, we evaluated Cten protein expression by immunohistochemistry. The association between Cten expression and patient survival was examined using Kaplan-Meier survival analysis, and univariate and multivariate Cox regression analyses were used to estimate the crude and adjusted hazard ratios.

**Results:**

Strong Cten expression was detected in 7%, 24%, 41%, and 46% of normal nevi, dysplastic nevi, primary melanoma, and metastatic melanoma samples, respectively, and Cten expression was found to be significantly higher in dysplastic nevi compared to normal nevi (P = 0.046), and in primary melanoma compared to dysplastic nevi (P = 0.003), but no difference was observed between metastatic and primary melanoma. Cten staining also correlated with AJCC stages (P = 0.015) and primary tumor thickness (P = 0.002), with Cten expression being induced in the transition from thin (<1mm) to thick (≥1mm) melanomas. Strong Cten expression was significantly associated with a worse 5-year overall (P = 0.008) and disease-specific survival (P = 0.004) for primary melanoma patients, and multivariate Cox regression analysis revealed that Cten expression was an independent prognostic marker for these patients (P = 0.038 for overall survival; P = 0.021 for disease-specific survival).

**Conclusion:**

Our findings indicate that induction of Cten protein expression is a relatively early event in melanoma progression, and that Cten has the potential to serve as a prognostic marker for primary melanoma patients.

## Background

Cutaneous malignant melanoma is a highly aggressive form of skin cancer with a steadily increasing rate of incidence in the non-Hispanic white population throughout the world [1-3], with the highest incidence rates observed in Australian and New Zealand males [1,2]. Although melanoma accounts for less than 5% of all skin cancers, it is responsible for the majority of all skin cancer-related deaths. Malignant melanoma is a highly invasive malignancy, and due to its high metastatic potential, the median survival for patients diagnosed with distant metastases is only 6-8 months, with an overall 5-year survival rate as low as 5-16% [3-5]. However, if the primary tumor is found and surgically removed before metastasis has occurred, the 5-year survival rate is approaching 100% [3,5], demonstrating the importance of early detection, diagnosis, and prognosis. When used in combination with traditional prognostic markers, proteins that are differentially expressed during tumorigenesis could help create more reliable prognoses. Yet, despite numerous tissue biomarkers having already been identified for melanoma, currently none are routinely used clinically to improve risk stratification [6], indicating a growing demand to identify dependable prognostic tissue markers for this disease.

C-terminal tensin-like protein (Cten) is a novel focal adhesion protein belonging to the tensin family of proteins together with tensin-1, tensin-2, and tensin-3, and is hence also sometimes referred to as tensin-4 [7]. All tensin family members contain C-terminal Src homology 2 (SH2) and phosphotyrosine-binding (PTB) domains, but unlike tensin1-3, which all contain an N-terminal actin binding domain (ABD), Cten does not [8]. The protein expression pattern of Cten appears to be highly tissue-dependent, with high expression levels found in the prostate, in which it was first identified a decade ago as a potential tumor suppressor, and placenta, with no, or relatively low, expression reported for all other normal tissues [8]. Since its initial discovery, several studies have examined the expression status of Cten in a number of cancers, with somewhat conflicting results. Whereas Cten appears to be down-regulated in prostate and kidney cancers [8,9], it has been found to be up-regulated and proposed to function as an oncogene in thymomas, lung, gastric, colorectal, breast, and pancreatic cancers [10-16]. The status of Cten expression in melanoma is currently unknown, and hence we were interested in examining this. 

Using immunohistochemical staining and tissue microarrays (TMAs) containing 29 normal nevi (NN), 88 dysplastic nevi (DN), 297 primary melanomas (PM), and 148 metastatic melanomas (MM), we examined the expression profile of Cten in melanoma progression, as well as the correlation between Cten expression and melanoma patient survival, and other clinicopathological characteristics. Our data showed that Cten was expressed significantly higher in dysplastic nevi compared to normal nevi, and in primary melanomas compared to dysplastic nevi, but that there was no difference between primary and metastatic melanomas, indicating that Cten expression is induced in the early stages of melanoma progression, rather than during metastasis. Furthermore, strong Cten expression was significantly associated with a poorer overall and disease-specific 5-year survival, and was an independent prognostic marker, for primary melanoma patients.

## Materials and Methods

### Ethics statement

All aspects of this study, including the use of human tissues and the waiver of patient consent, were performed in accordance with the Declaration of Helsinki guidelines, as approved by the Clinical Research Ethics Board of the University of British Columbia, Vancouver, Canada.

### TMA construction

748 formalin-fixed, paraffin-embedded tissues were obtained from Vancouver General Hospital, Department of Pathology, between 1992 and 2009. Tissues with insufficient tumor cells or lost cores were excluded from the study, resulting in a total of 562 tissues available for evaluation, including 29 normal nevi, 88 dysplastic nevi, 297 primary melanomas, and 148 metastatic melanomas. The TMAs were assembled using a tissue-array instrument (Beecher Instruments, Silver Spring, MD), and duplicate 0.6mm thick tissue cores were taken from each biopsied tissue. Multiple 4μm sections were cut using a Leica microtome (Leica Microsystems Inc., Bannockburn, IL), and transferred to adhesive-coated slides using standard procedures. From each TMA set, one section was stained with hematoxylin and eosin as per standard protocol, and the remaining sections were reserved for immunohistochemical staining.

### Immunohistochemistry of TMA

The TMA slides were deparaffinized by heating at 55°C for 20 minutes followed by three 5-minute washes with xylene, and rehydrated by consecutive 5-minute washes in 100%, 95% and 80% ethanol, and distilled water. Antigen retrieval was accomplished by heating the samples at 95°C in 10 mM sodium citrate at pH 6.0 for 30 minutes. Endogenous peroxidase activity was blocked by incubation of the slides in 3% hydrogen peroxide for 30 minutes. The tissues were blocked with Dako antibody diluent (Dako Diagnostics, Glostrup, Denmark) for 30 minutes to prevent non-specific binding, followed by incubation with a primary mouse monoclonal anti-Cten antibody (clone 684524, 1:50 dilution, R&D Systems, Minneapolis, MN) overnight at 4°C. Incubation in the antibody diluent without the primary antibody served as the negative control. A small subset of metastatic melanoma samples (n=10) was stained with a second primary monoclonal anti-Cten antibody (clone SP83, 1:100 dilution, Abnova, Walnut, CA) to determine if the results obtained from our main study were reproducible. Next, the samples were incubated with a universal biotinylated secondary antibody followed by streptavidin-HRP (Dako Diagnostics) for 30 minutes each, and developed using 3,3’-diaminobenzidine substrate (Vector Laboratories, Burlington, ON, Canada). Lastly, hematoxylin counterstaining was performed to visualize the nuclei, and the slides were dehydrated and sealed with cover slips.

### Evaluation of immunostaining

Cten staining was evaluated and scored based on intensity of staining (0-3) and percentage of Cten-positive cells [1 (0-25%; 2 (26-50%); 3 (51-75%); and 4 (76-100%)] by two independent observers, who were blinded to other clinical data. The level of staining was calculated by multiplying the intensity score and the percentage of staining, and was identified as: negative (0); weak (1-3); moderate (4-6); and strong (8-12). In the event of two duplicate cores having different staining, the higher score of the two was used for statistical analysis. Cten expression was defined as being either negative to moderate (neg-mod; 0-6) or strong (8-12), based on the level of staining.

### Statistical analysis

Differences in demographic and clinicopathological characteristics, and Cten expression between subgroups were evaluated by χ^2^ tests (degrees of freedom (df) = 1, unless otherwise stated). Kaplan-Meier and log-rank tests were used to evaluate the correlation between Cten expression and overall and disease-specific 5-year survival outcomes for melanoma patients. Univariate and multivariate Cox regression analyses were used to determine the crude and adjusted hazard ratios, respectively, and their 95% confidence intervals. For all tests, a P-value <0.05 was considered significant. SPSS version 16.0 (SPSS Inc., Chicago, IL) software was used for all analyses. 

## Results

### Clinicopathological features of TMAs

TMAs containing 29 normal nevi, 88 dysplastic nevi, 297 primary melanoma, and 148 metastatic melanoma were evaluated for Cten staining. Figure S1 shows the inclusion and exclusion of patient samples. For the 445 melanoma cases, there were 263 males and 182 females, with a median age of 60 (ranging between 7 and 95 years). A total of 170 tumors were classified as AJCC stage I, 127 as stage II, 62 as stage III, and 83 as stage IV melanomas. Three samples lacked information regarding AJCC stage. For primary melanoma, the tumors were sub-classified as acral lentiginous (n=9), desmoplastic (n=11), lentigo maligna (n=63), nodular (n=44), superficial spreading (n=109) and other (unclassified, spitz-like, and nevoid, n=61) melanomas. Of these, 83 tumors were biopsied from the head and neck, and 211 from other, sun-protected sites. 99 of the primary tumors were <1mm or *in situ*, 73 tumors were 1-<2mm, 64 were 2-4mm, and 61 were >4mm thick. Ulceration was present in 53 cases. For metastatic melanoma, 64 tumors were cutaneous, 55 were biopsied from lymph nodes, and 26 were obtained from visceral organs, while 3 samples lacked information about the location of the metastatic deposit (Table 1).

**Table 1:** Cten staining and clinicopathological characteristics of 445 melanoma patients.

	**Cten staining**			
**Variables**	**Neg-Mod**	**Strong**	**Total**	**P-value^1^**
*Primary Melanoma (n=297)*				
Age				
≤60	85 (58.2%)	61 (41.8%)	146	0.808
>60	90 (59.6%)	61 (40.4%)	151	
Sex				
Male	101 (61.6%)	63 (38.4%)	164	0.300
Female	74 (55.6%)	59 (44.4%)	133	
Tumor thickness (mm)				
<1.0	71 (71.7%)	28 (28.3%)	99	0.002^2^
1.0 - <2.0	40 (54.8%)	33 (45.2%)	73	
2.0 - 4.0	36 (56.25%)	28 (43.75%)	64	
>4.0	29 (47.5%)	32 (52.5%)	61	
Ulceration				
Present	30 (56.6%)	23 (43.4%)	53	0.705
Absent	145 (59.4%)	99 (40.6%)	244	
Tumor subtype				
Acral Lentiginous	6 (66.7%)	3 (33.3%)	9	
Desmoplastic	6 (54.5%)	5 (45.5%)	11	
Lentigo Maligna	44 (69.8%)	19 (30.2%)	63	
Nodular	17 (38.6%)	27 (61.4%)	44	0.003^3^
Superficial Spreading	60 (55.0%)	49 (45.0%)	109	
Other^4^	43 (70.5%)	18 (29.5%)	61	0.045^5^
Location^6^				
Sun-protected	112 (53.1%)	99 (46.9%)	211	0.001
Sun-exposed	61 (73.5%)	22 (26.5%)	83	
*Metastatic Melanoma (n=148)*				
Age				
≤60	47 (54.7%)	39 (45.3%)	86	0.865
>60	33 (53.2%)	29 (46.8%)	62	
Sex				
Male	53 (53.5%)	46 (46.5%)	99	0.858
Female	27 (55.1%)	22 (44.9%)	49	
AJCC stage				
I	110 (64.7%)	60 (35.3%)	170	0.015^7^
II	65 (51.2%)	62 (48.8%)	127	
III	30 (48.4%)	32 (51.6%)	62	
IV	49 (59.0%)	34 (41.0%)	83	
Location				
Cutaneous	34 (53.1%)	30 (46.9%)	64	0.226^8^
Lymph nodal	27 (49.1%)	28 (50.9%)	55	
Visceral	18 (69.2%)	8 (30.8%)	26	

AJCC indicates American Joint Committee on Cancer.

^1^ χ^2^ test, df = 1 unless otherwise stated.

^2^ Tumors <1.0mm thick vs. all other tumors.

^3^ Nodular melanoma vs. all other subtypes.

^4^ Other: Unspecified subtypes (n=58), spitz-like melanoma (n=1) and nevoid melanoma (n=2).

^5^ Other melanomas vs. all other subtypes.

^6^ Sun-protected locations: back, trunk, arms, hands, legs, feet, and vulva; Sun-exposed sites: head and neck. Cases with unspecified location (n=3) were excluded from analysis.

^7^ AJCC stage I vs. stages II-IV. Samples with unspecified AJCC stages (n=3) were excluded from analysis.

^8^ Df = 2. Samples lacking information about the location of metastasis (n=3) were excluded from the study

### Cten expression is increased during melanoma development

**Figure 1:**
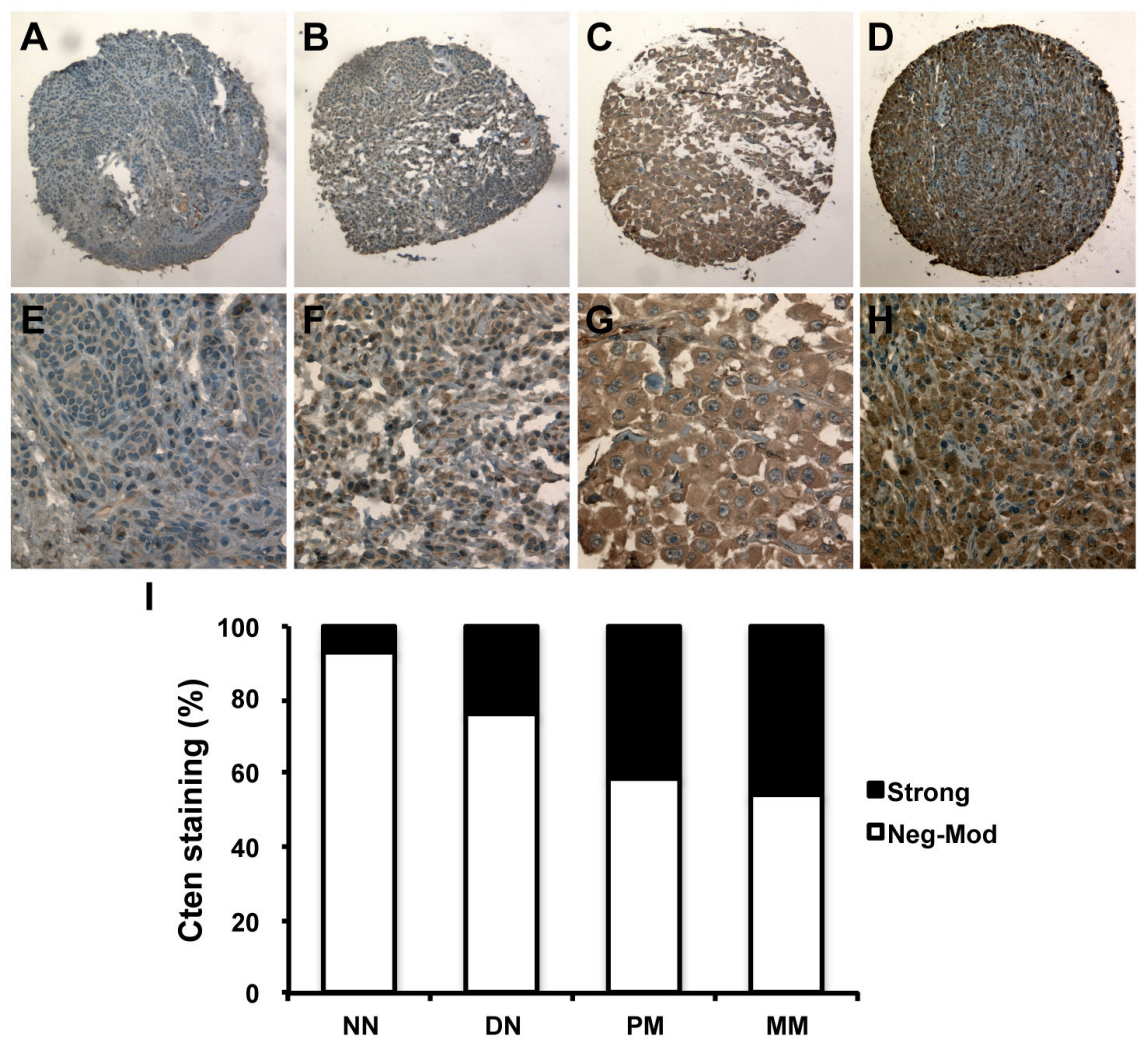
Representative images of Cten protein expression at 100x (A-D) and 400x magnification (E-H). (A, E) Weak Cten staining in normal nevi (NN). (B, F) Moderate Cten staining in dysplastic nevi (DN). (C, G) Strong Cten staining in primary melanoma (PM). (D, H) Strong Cten staining in metastatic melanoma (MM). (I) Correlation between Cten expression and melanoma progression.

As seen in Figure 1A-H, Cten was expressed in the cytoplasm at all stages of melanocytic lesions, and staining with a second primary anti-Cten antibody in a small subset of tumors supported these results (Figure S2). Strong Cten staining was observed in 7%, 24%, 41%, and 46% of normal nevi, dysplastic nevi, primary melanoma, and metastatic melanoma samples, respectively. Cten protein expression was found to be significantly higher in dysplastic nevi compared to normal nevi (P = 0.046, χ^2^ test), in primary melanoma compared to dysplastic and normal nevi (P = 0.003 and < 0.001, respectively, χ^2^ tests), and in metastatic melanoma compared to dysplastic and normal nevi (P < 0.001 for both, χ^2^ tests), but no significant difference between metastatic and primary melanoma was observed (P = 0.328, χ^2^ test, Figure 1I). 

### Correlation between Cten expression and clinicopathological characteristics

**Figure 2:**
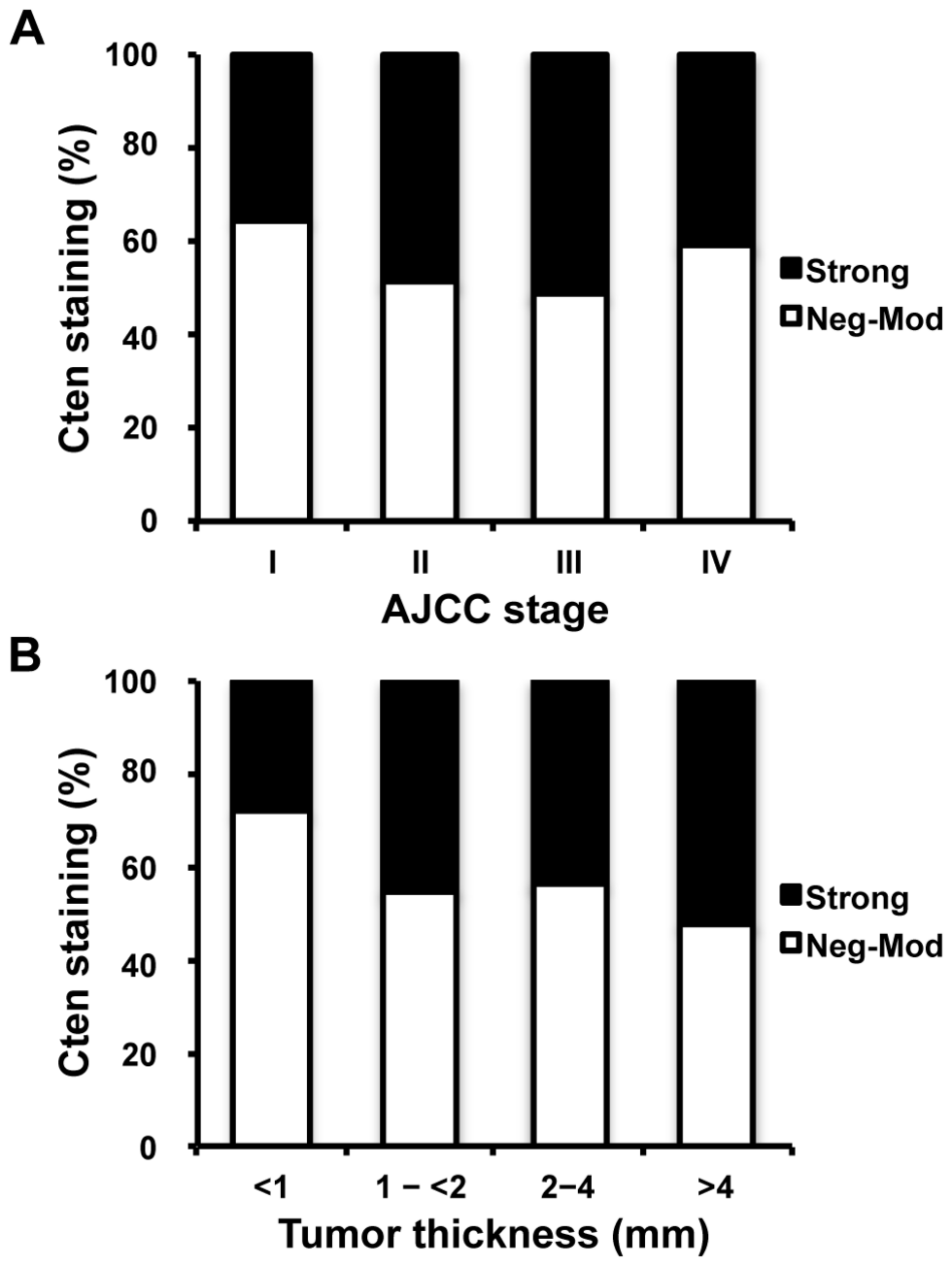
Cten expression correlates with AJCC stages and tumor thickness. (A) Cten staining was significantly increased in AJCC stage II-IV tumors compared to AJCC stage I tumors (P = 0.015, χ^2^ test). (B) Cten expression was significantly higher in invasive, thick tumors ≥1mm compared to *in* *situ* and thin tumors <1mm (P = 0.002, χ^2^ test).

Strong Cten expression was observed in 35% of AJCC stage I melanomas compared to 47% of melanomas belonging to AJCC stages II-IV (P = 0.015, χ^2^ test, Figure 2A). For primary melanoma, Cten staining was found to be significantly higher in tumors ≥1mm thick (47% strong staining), compared to *in situ* and thin tumors <1mm (28% strong staining, P = 0.002, χ^2^ test, Figure 2B). Interestingly, we found that Cten was expressed at comparable levels in dysplastic nevi (24% strong staining, Figure 1I) and primary tumors <1mm thick (28%, P = 0.493, χ^2^ test), and in primary tumors ≥1mm thick (47% strong staining) and metastatic melanomas (46%, Figure 1I, P = 0.850, χ^2^ test).

Strong Cten expression was detected in 61% of nodular melanomas compared to in only 37% of all other primary melanoma subtypes (P = 0.003, χ^2^ test), and it was also found to be significantly lower expressed in other, mainly unclassified, melanomas (P = 0.045, χ^2^ test). A significant difference in Cten expression was furthermore observed between tumors found at sun-protected sites (47% strong staining) compared to tumors from the head and neck (27%, P = 0.001, χ^2^ test), whereas no correlation was seen for Cten and ulceration status or primary melanoma patient age and sex. Additionally, no correlation between Cten expression and patient age and sex, or tumor location for metastatic melanoma patients was detected (Table 1). 

### Correlation between Cten expression and 5-year survival of melanoma patients

**Figure 3:**
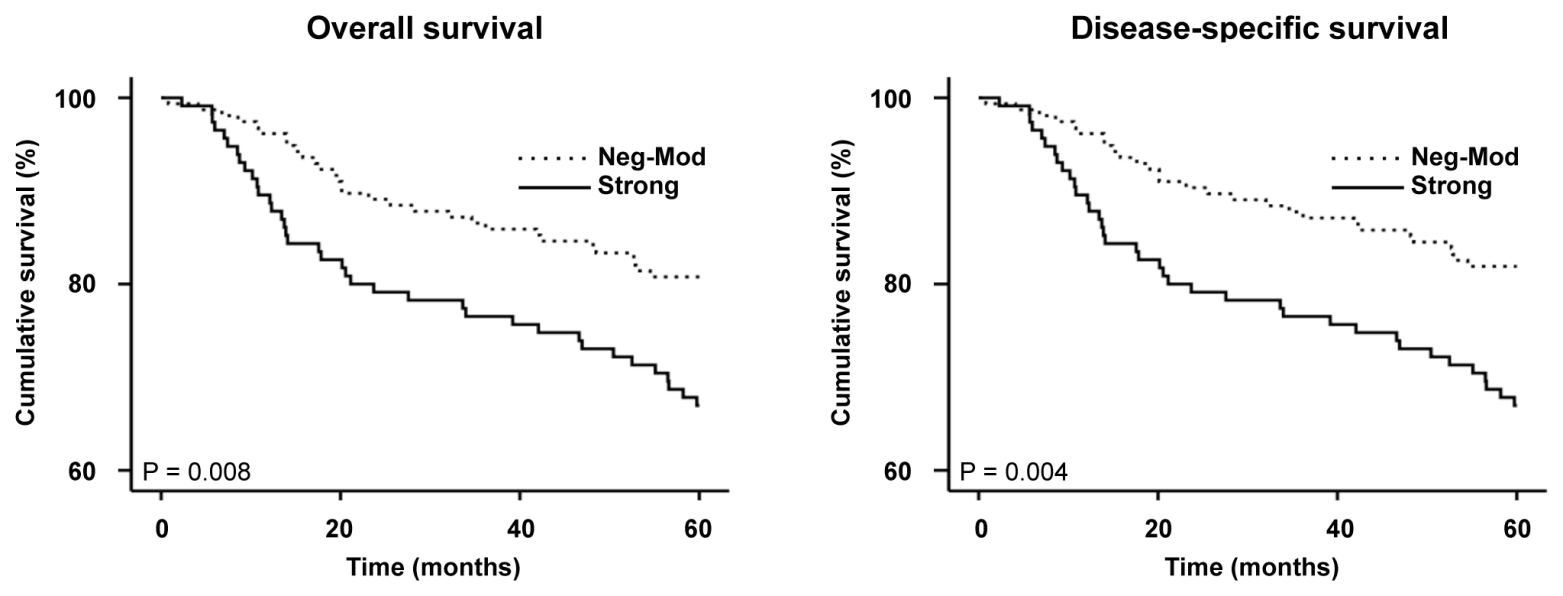
Kaplan-Meier analysis for the correlation between Cten expression and 5-year survival in 271 primary melanoma patients. Strong Cten expression was significantly associated with a worse overall and disease-specific 5-year survival for primary melanoma patients (P = 0.008 and 0.004, respectively, log-rank tests).

A total of 418 melanoma patients (271 primary melanoma and 147 metastatic melanoma patients) had complete follow-up and clinical information (Figure S1). Survival times were calculated as time from diagnoses to last follow-ups or death. Kaplan-Meier analyses revealed that Cten expression was significantly associated with the overall (P = 0.008) and disease-specific (P = 0.004) 5-year survival for primary melanoma patients (log-rank tests, Figure 3). As expected, no significant correlation between Cten expression and the overall and disease-specific 5-year survival of all melanoma patients (P = 0.079 and 0.072, respectively) or metastatic melanoma patients (P = 0.434 and 0.367, respectively) was observed (data not shown). 

**Figure 4:**
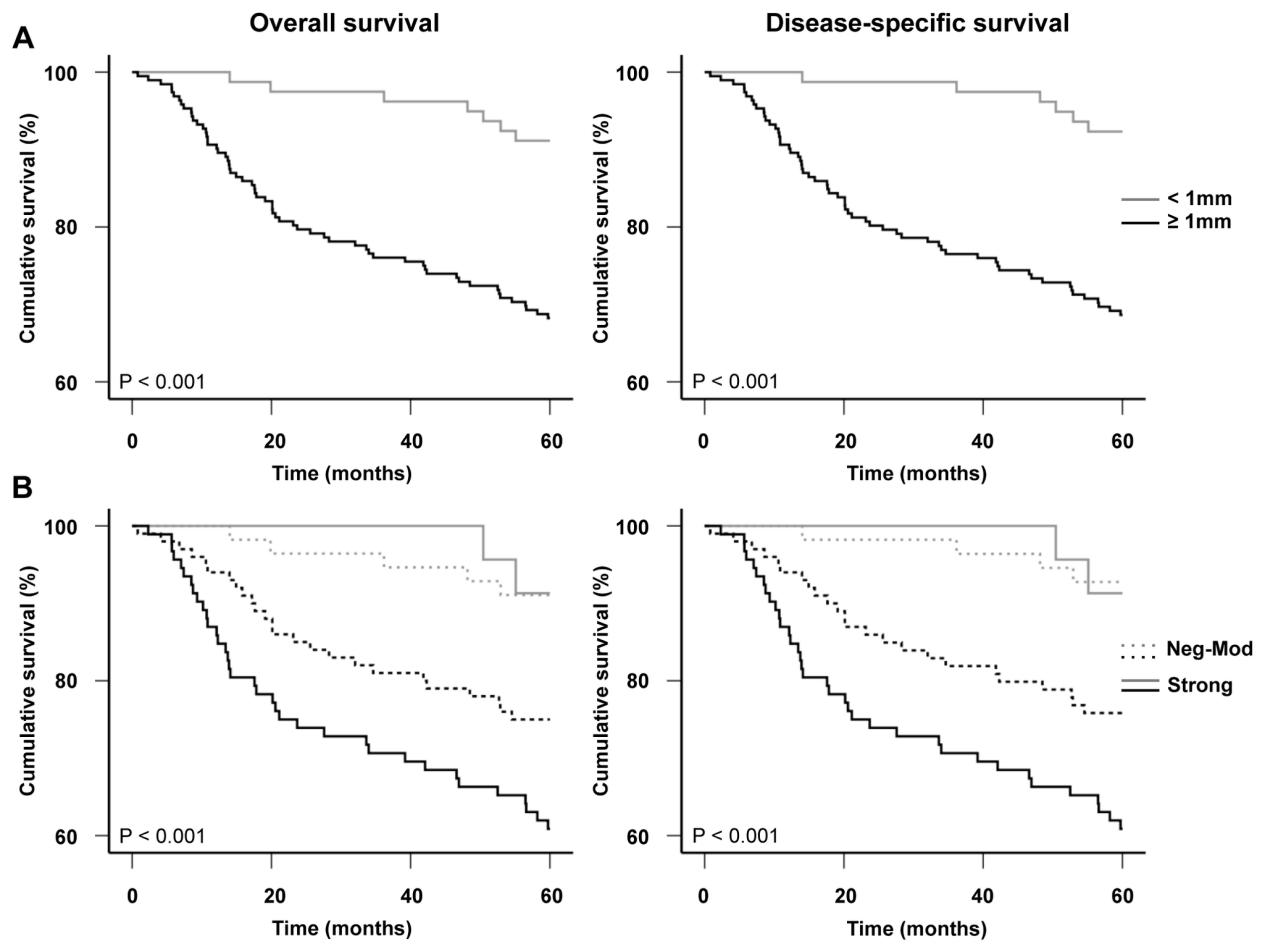
Kaplan-Meier analyses for the correlations between primary tumor thickness, Cten expression, and patient survival. (A) Tumors ≥1mm thick (n=192) were significantly associated with a poorer overall and disease-specific survival for primary melanoma patients compared to tumors <1mm thick (n=79, P < 0.001 for both, log-rank tests). (B) Strong Cten staining was significantly associated with a poorer overall and disease-specific 5-year survival for patients with tumors ≥1mm thick (black lines, P = 0.031 and 0.022, respectively) but not with tumors <1mm thick (gray lines, P = 0.939 and 0.857, respectively). A significant difference was also observed between tumors <1mm thick with neg-mod Cten expression and tumors ≥1mm thick with neg-mod (P = 0.014 and 0.009, respectively) or strong Cten expression (P = 0.001 and <0.001, respectively), and between tumors <1mm thick with strong Cten expression and tumors ≥1mm thick with strong Cten expression (P = 0.007 for both), but not between tumors <1mm thick with strong Cten expression and tumors ≥1mm with neg-mod Cten expression (P = 0.085 and 0.098, respectively, P < 0.001 overall, log-rank tests).

Since Cten expression appeared to be induced in the transition from thin (<1mm) to thick (≥1mm) melanoma (Figure 2B), we next examined whether there were any differences in patient survival between these groups. Figure 4A shows the association between tumor thickness and primary melanoma patient survival (P < 0.001 for both overall and disease-specific 5-year survival). When Cten expression was included in the analysis, we observed a significant difference in the 5-year survival of melanoma patients with tumors ≥1mm thick, with strong Cten expression being associated with a poorer survival outcome (P = 0.031 and 0.022 for overall and disease-specific survival, respectively), but no correlation between Cten and patient survival was observed for tumors <1mm thick (P = 0.939 and 0.857 for overall and disease-specific survival, respectively, P < 0.001 overall, log-ranks tests, Figure 4B). It is worth noting however, that the number of deaths in this category was very low, so no firm conclusions about the association between Cten and patient survival can be made for this group without repeating the experiment in a larger study.

### Cten is an independent prognostic marker for primary melanoma patients

Univariate Cox proportional hazard regression analysis showed that strong Cten expression was a significant prognostic factor for primary melanoma patients ((HR, 1.89, 95% CI, 1.17-3.05, P = 0.009) for overall survival, and (HR, 2.03, 95% CI, 1.24-3.30, P = 0.005) for disease-specific survival, Table 2). Lastly, we used multivariate Cox regression analysis to examine if Cten was also an independent prognostic marker for primary melanoma patient survival. Sex, age, tumor thickness, ulceration status, tumor location, and Cten expression were included in the analysis, and the results indicated that Cten was an adverse independent prognostic factor for the overall (HR, 1.69, 95% CI, 1.03-2.77, P = 0.038) and disease-specific 5-year survival of primary melanoma patients (HR, 1.82, 95% CI, 1.10-3.01, P = 0.021, Table 3).

**Table 2:** Univariate Cox regression analysis on 5-year overall and disease-specific survival of 271 primary melanoma patients.

**Variables**	**Patients**	**Overall survival**				**Disease-specific survival**			
		**Deaths**	**Death Rate**	**HR (95% CI)**	**P-value^1^**	**Deaths**	**Death Rate**	**HR (95% CI)**	**P-value^1^**
Age									
≤60	134 (49.4%)	24	17.9%	1.00	0.007	22	16.4%	1.00	0.003
>60	137 (50.6%)	44	32.1%	1.99 (1.21-3.27)		44	32.1%	2.17 (1.30-3.62)	
Sex									
Male	148 (54.6%)	36	24.3%	1.00	0.726	35	23.6%	1.00	0.743
Female	123 (45.4%)	32	26.0%	1.09 (0.68-1.75)		31	25.2%	1.08 (0.67-1.76)	
Thickness (mm)									
<1.00	79 (29.2%)	7	8.9%	1.00	<0.001	6	7.6%	1.00	<0.001
≥1.00	192 (70.8%)	61	31.8%	4.23 (1.93-9.24)		60	31.3%	4.85 (2.10-11.23)	
Ulceration									
Absent	221 (81.5%)	39	17.6%	1.00	<0.001	37	16.7%	1.00	<0.001
Present	50 (18.5%)	29	58.0%	4.56 (2.81-7.38)		29	58.0%	4.81 (2.95-7.85)	
Location^2^									
Sun-protected	198 (73.9%)	51	25.8%	1.00	0.602	49	24.7%	1.00	0.703
Sun-exposed	70 (26.1%)	16	22.9%	0.86 (0.49-1.51)		16	22.9%	0.90 (0.51-1.58)	
Cten staining									
Neg-Mod	156 (57.6%)	30	21.4%	1.00	0.009	28	19.8%	1.00	0.005
Strong	115 (42.4%)	38	33.0%	1.89 (1.17-3.05)		38	33.0%	2.03 (1.24-3.30)	

^1^ Log-rank test.

^2^ Sun-protected locations: back, trunk, arms, hands, legs, feet, and vulva; Sun-exposed sites: head and neck. Cases with unspecified location (n=3) were excluded from analysis.

Abbreviations: HR, hazard ratio; CI, confidence interval.

**Table 3:** Multivariate Cox regression analysis on 5-year overall and disease-specific survival of 271 primary melanoma patients.

**Variables^1^**	**Overall survival**					**Disease-specific survival**				
	**β^2^**	**SE**	**HR**	**95.0% CI**	**P-value^3^**	**β^2^**	**SE**	**HR**	**95.0% CI**	**P-value^3^**
Sex	0.034	0.247	1.03	0.64-1.68	0.891	0.028	0.251	1.03	0.63-1.68	0.912
Age	0.380	0.265	1.46	0.87-2.46	0.152	0.447	0.272	1.56	0.92-2.67	0.100
Thickness	0.934	0.420	2.55	1.12-5.80	0.026	1.040	0.449	2.83	1.17-6.82	0.021
Ulceration	1.185	0.262	3.27	1.96-5.47	<0.001	1.212	0.265	3.36	2.00-5.64	<0.001
Location	0.042	0.299	1.04	0.58-1.88	0.889	0.096	0.301	1.10	0.61-1.99	0.749
Cten	0.524	0.253	1.69	1.03-2.77	0.038	0.596	0.258	1.82	1.10-3.01	0.021

^1^ Coding of variables: Age was coded as 1 (≤60 years) and 2 (>60 years); sex was coded as 1 (male) and 2 (female); tumor thickness was coded as 1 (<1mm) and 2 (≥1mm); ulceration was coded as 1 (absent) and 2 (present); location was coded as 1 (sun-protected) and 2 (sun-exposed); Cten was coded as 1 (negative-moderate expression) and 2 (strong expression).

^2^ β = regression coefficient.

^3^ Log-rank test.

Abbreviations: SE, standard error; HR, hazard ratio; CI, confidence interval.

## Discussion

Even though Cten was first identified as a potential tumor suppressor in prostate cancer [8], it has since been reported to function as an oncogene in numerous other cancers, but the mechanisms behind this remain controversial [10-16]. Since the status of Cten in melanoma is currently unknown, we here investigated the protein expression of Cten in a large number of human melanocytic lesions as a first step to elucidate its role in melanomagenesis. We detected exclusively cytoplasmic Cten staining (Figure 1A-H), and despite of Cten originally being described as a focal adhesion protein [8], we did not distinguish a clear focal adhesion pattern. Staining with a second primary anti-Cten antibody in a small subset of tissues yielded comparable results, and supported these findings (Figure S2). Similarly, in breast and colorectal cancers, the immunohistochemical staining pattern of Cten has been described as homogenous cytoplasmic, with no samples displaying focal adhesion staining [15,17]. Nuclear expression of Cten was also reported in a fraction of colorectal cancer samples [14,17], but it is likely that this discrepancy represents yet another tissue-specific characteristic of Cten, since to our knowledge, this has not been reported in any other types of cancer, and does not seem to have any clinical significance [17]. 

Cten protein expression was significantly increased in the progression from melanocytic nevi to primary melanoma, but no difference in Cten staining between primary and metastatic melanomas was detected in this study (Figure 1I). We next separated the primary tumors into four groups based on their thickness (Figure 2B), and found that while Cten was expressed at comparable levels in primary tumors <1mm thick (28% strong staining, Figure 2B) and dysplastic nevi (24% strong staining, Figure 1I), Cten expression was significantly increased in tumors ≥1mm thick (45%, 44% and 53% strong staining for tumors 1-<2mm, 2-4mm, and >4mm thick, respectively, Figure 2B). Likewise, the expression level of Cten in metastatic melanoma samples (46% strong staining, Figure 1I) was similar to that of primary tumors ≥1mm thick (47% strong staining, Figure 2B), and no significant difference was seen between cutaneous, lymph nodal or visceral metastatic melanomas (Table 1), or between AJCC stage III and stage IV melanomas (Figure 2A), indicating that Cten expression is likely induced during the transition from thin (<1mm) to thick (≥1mm) melanomas rather than during metastasis. Accordingly, Cten expression was significantly higher in tumors classified as AJCC stages II-IV compared to AJCC stage I tumors (Figure 2A). 

To investigate the role of Cten in melanoma patient survival, we constructed Kaplan-Meier survival curves. Analyses showed that strong Cten expression significantly correlated with a poorer overall and disease-specific 5-year survival for primary melanoma patients (P = 0.008, and 0.004, respectively, Figure 3). These data are similar to what has been previously observed in gastric, breast and colorectal cancers [12,15,17]. Subsequent multivariate Cox regression analysis showed that strong Cten expression, when adjusted to sex, age, tumor thickness, location and status of ulceration, was an adverse independent prognostic factor for the overall (HR, 1.69, 95% CI, 1.03-2.77, P = 0.038) and disease-specific (HR, 1.82, 95% CI, 1.10-3.01, P = 0.021) 5-year survival of primary melanoma patients (Table 3). While there are already numerous tissue biomarkers identified for melanoma [6], many of these strongly correlate with Breslow’s thickness – one of the most significant prognostic markers for primary melanoma patient survival [18]. Cten is no exception, and we here showed that Cten was significantly stronger expressed in tumors ≥1mm compared to tumors <1mm thick (Figure 2B), and that strong Cten expression was significantly associated with a poorer survival specifically in patients with tumors ≥1mm thick (Figure 4B). However, our survival analysis also showed that even though there was a trend, there was no significant difference in patient survival between tumors <1mm thick with strong Cten staining and tumors ≥1mm thick with negative-moderate Cten staining (Figure 4B), and by including the same cut-offs for tumor thickness into our multivariate analysis (Table 3), we demonstrated that Cten was a significant prognostic factor for primary melanoma patient survival, independent of tumor thickness. This, together with the fact that Cten appears to have a highly restricted expression pattern in most normal tissues [8], suggests that it could have the potential to one day be used clinically as a prognostic marker.

While it is currently unclear exactly how Cten is activated and regulated, there seem to be multiple pathways involved. Intriguingly, strong Cten expression has been associated with KRAS/BRAF mutations in colorectal cancer, and this association was confirmed as being functionally relevant in colorectal and pancreatic cancers, where Cten has been identified as a downstream target of KRAS signaling [19]. Unlike in colorectal cancer however, where KRAS mutations have been described in 34-42% of all tumors, KRAS mutations in melanoma are rarely observed [20-23]. BRAF mutations on the other hand, especially the V600E point mutation, have been observed in over half of all melanomas [24,25], and if there is a correlation between BRAF mutations and Cten expression in melanoma as well, and if Cten is in fact an oncoprotein, it could provide an attractive additional target for therapeutic intervention. In support of these findings, Hung et al. (2013) recently reported that Cten expression was induced by a number of growth factors, including EGF and FGF2, via the MAPK pathway in prostate and colon cancer cell lines [26]. However, whether this is also the case in melanoma, or whether Cten induction is the result of other signaling cascades, will have to be examined in more detail before any conclusions about the regulation and function of Cten in melanomagenesis can be drawn.

In summary, we here found that Cten expression was significantly increased in the progression from nevi to primary melanoma, with the most drastic increases in Cten expression being observed between normal and dysplastic nevi, and between primary tumors <1mm thick and tumors ≥1mm thick, indicating that Cten induction is a relatively early event in melanoma progression. Moreover, we found that strong Cten expression was associated with a significantly worse 5-year survival, and was an adverse independent prognostic marker, for primary melanoma patients. Although this study would have to be repeated with an even larger set of samples for any firm conclusions to be drawn, these preliminary results support the use of Cten as a potential prognostic marker for primary melanoma patients, and warrant for further studies into the function of this protein in melanomagenesis.

## Supporting Information

Figure S1 **CONSORT diagram for melanoma patient inclusion and exclusion.**
(DOCX)

Figure S2 **Immunohistochemical staining using two different primary antibodies in a subset of metastatic melanoma samples known to vary widely in Cten expression (at 100x).** (A-E) Staining with the primary mouse monoclonal anti-Cten antibody at 1:50 dilution (clone 684524, R&D Systems, Minneapolis, MN) in five (5) representative cores. (F-J) Staining of matching samples with a primary rabbit monoclonal anti-Cten antibody at 1:100 dilution (clone SP83, Abnova, Walnut, CA).
(TIF)
