# Supplementary figures and images for: C-Terminal Tensin-Like Protein Is a Novel Prognostic Marker for Primary Melanoma Patients
Source: PLoS One. 2013 Nov 7;8(11):e80492. doi: 10.1371/journal.pone.0080492 (PMC3820571; doi:10.1371/journal.pone.0080492)

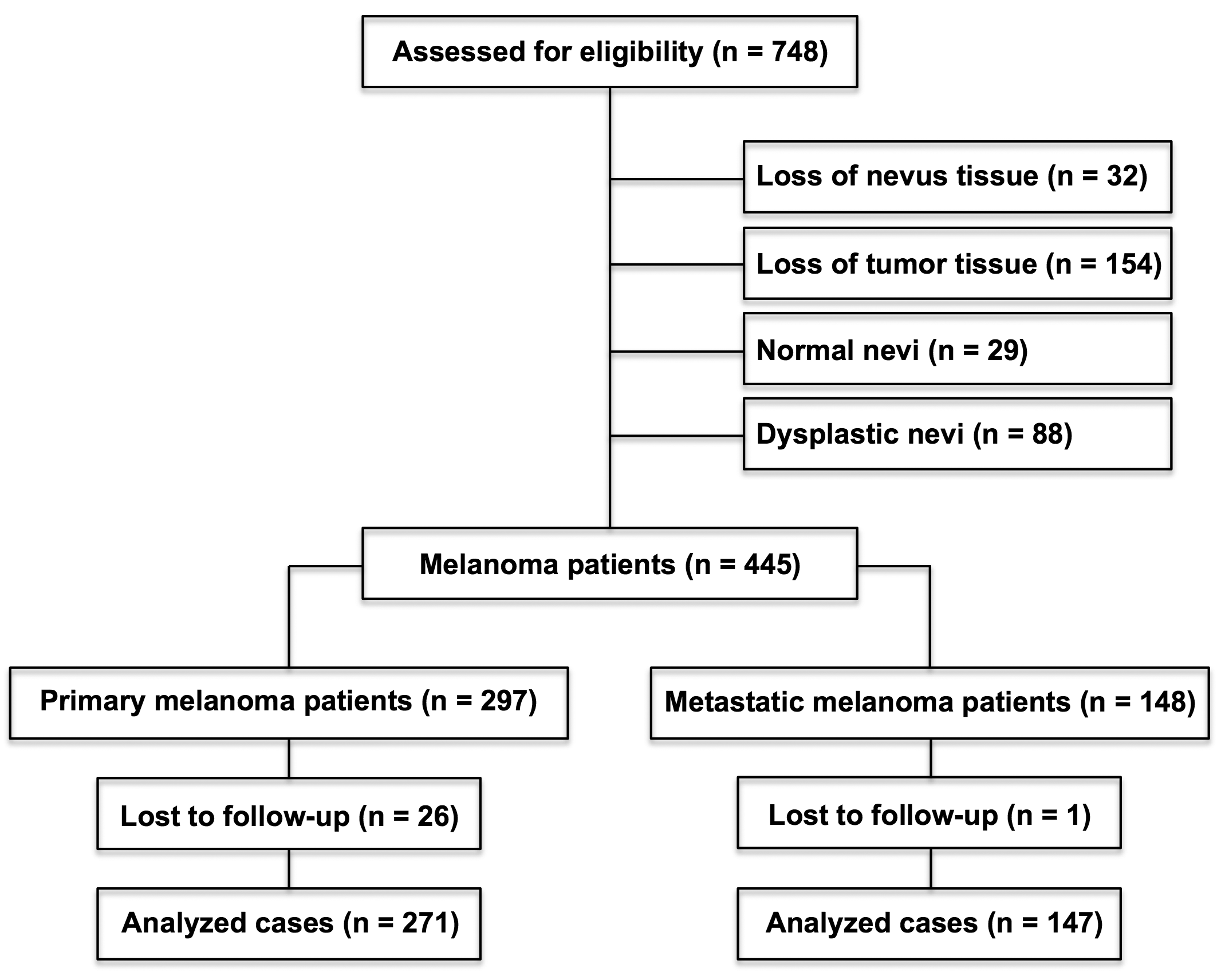

Supplement: Figure S1 — CONSORT diagram for melanoma patient inclusion and exclusion. (DOCX) [file pone.0080492.s001.docx]

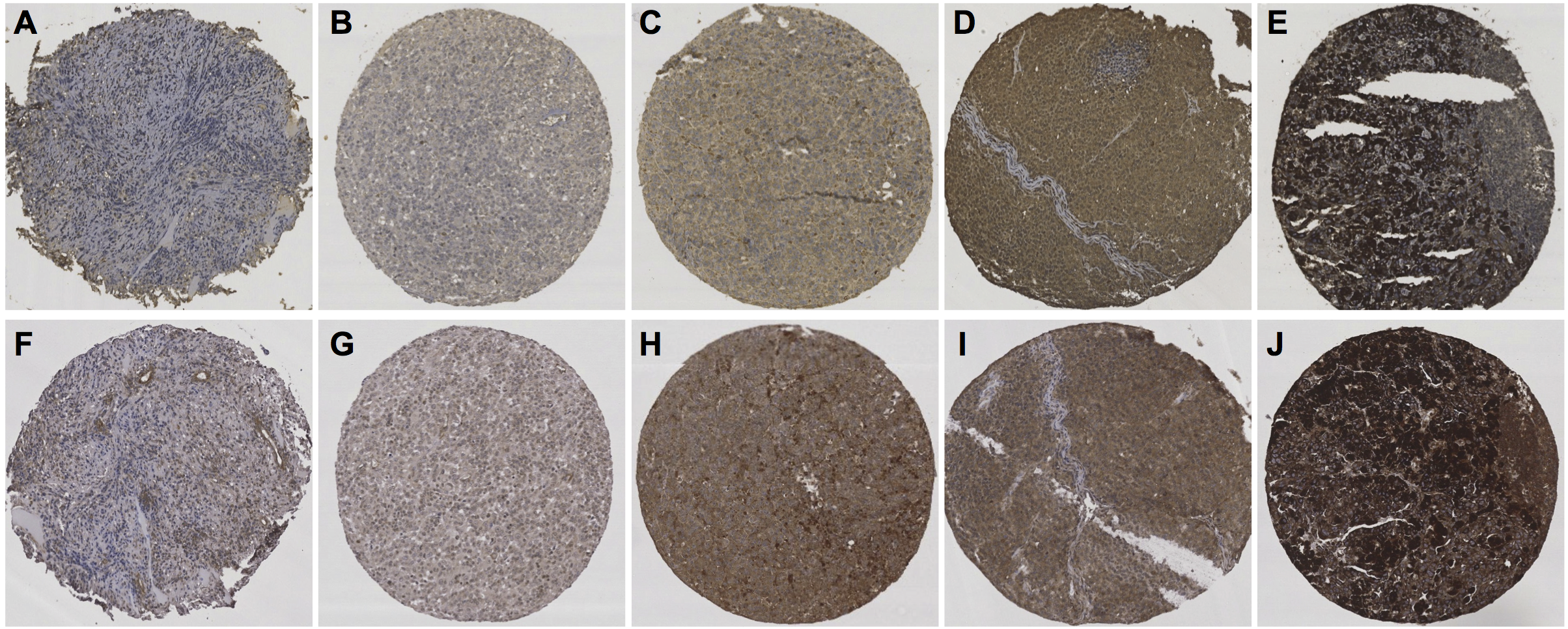

Supplement: Figure S2 — Immunohistochemical staining using two different primary antibodies in a subset of metastatic melanoma samples known to vary widely in Cten expression (at 100x). (A-E) Staining with the primary mouse monoclonal anti-Cten antibody at 1:50 dilution (clone 684524, R&D Systems, Minneapolis, MN) in five (5) representative cores. (F-J) Staining of matching samples with a primary rabbit monoclonal anti-Cten antibody at 1:100 dilution (clone SP83, Abnova, Walnut, CA). (TIF) [file pone.0080492.s002.tif]
